# Structural variants are a major source of gene expression differences in humans and often affect multiple nearby genes

**DOI:** 10.1101/gr.275488.121

**Published:** 2021-12

**Authors:** Alexandra J. Scott, Colby Chiang, Ira M. Hall

**Affiliations:** 1 McDonnell Genome Institute, Washington University School of Medicine, St. Louis, Missouri 63108, USA;; 2 Department of Medicine, Washington University School of Medicine, St. Louis, Missouri 63110, USA;; 3 Department of Genetics, Yale University School of Medicine, New Haven, Connecticut 06510, USA

## Abstract

Structural variants (SVs) are an important source of human genome diversity, but their functional effects are poorly understood. We mapped 61,668 SVs in 613 individuals from the GTEx project and measured their effects on gene expression. We estimate that common SVs are causal at 2.66% of eQTLs, a 10.5-fold enrichment relative to their abundance in the genome. Duplications and deletions were the most impactful variant types, whereas the contribution of mobile element insertions was small (0.12% of eQTLs, 1.9-fold enriched). Multitissue analysis of eQTLs revealed that gene-altering SVs show more constitutive effects than other variant types, with 62.09% of coding SV-eQTLs active in all tissues with eQTL activity compared with 23.08% of coding SNV- and indel-eQTLs. Noncoding SVs, SNVs and indels show broadly similar patterns. We also identified 539 rare SVs associated with nearby gene expression outliers. Of these, 62.34% are noncoding SVs that affect gene expression but have modest enrichment at regulatory elements, showing that rare noncoding SVs are a major source of gene expression differences but remain difficult to predict from current annotations. Both common and rare SVs often affect the expression of multiple genes: SV-eQTLs affect an average of 1.82 nearby genes, whereas SNV- and indel-eQTLs affect an average of 1.09 genes, and 21.34% of rare expression-altering SVs show effects on two to nine different genes. We also observe significant effects on rare gene expression changes extending 1 Mb from the SV. This provides a mechanism by which individual SVs may have strong or pleiotropic effects on phenotypic variation.

Structural variants (SVs) are a diverse class of genetic variation that include copy number variants (CNVs), mobile element insertions (MEIs), and balanced rearrangements at least 50 bp in length. Although SVs are relatively rare compared with single-nucleotide variants (SNVs) and small insertion or deletion (indel) variants, their size and diversity mean that SVs can disrupt protein-coding genes and genomic regulatory elements through diverse mechanisms. Furthermore, SVs often have more severe consequences compared with smaller variants, and previous studies have found that SVs have an outsized impact on human gene expression compared with their relative abundance in the genome (Stranger et al. 2007; Sudmant et al. 2015; Chiang et al. 2017). SVs have also been implicated in the biology of human diseases such as autism spectrum disorder (Sebat et al. 2007; Weiss et al. 2008; Turner et al. 2017; Brandler et al. 2018) and schizophrenia (International Schizophrenia Consortium 2008; Walsh et al. 2008; McCarthy et al. 2009; Marshall et al. 2017). However, SVs are difficult to detect from short-read DNA sequencing data and are often excluded from complex trait association studies.

Advances in high-throughput sequencing technologies that have allowed for widespread use of whole-genome sequencing (WGS), combined with advances in scaling SV detection algorithms, mean that comprehensive studies of all forms of genetic variation are now possible for large human cohorts. Recent studies of SV in large, deeply sequenced human cohorts have found that SVs account for 4.0%–11.2% of rare high-impact coding alleles (Abel et al. 2020) and are responsible for 25%–29% of rare protein-truncating events per genome (Collins et al. 2020). However, few studies to date have examined the functional effects of SV on gene expression, and these studies are limited to relatively small cohort sizes or only a few tissue types with available gene expression data (Sudmant et al. 2015; Chiang et al. 2017; Han et al. 2020; Jakubosky et al. 2020).

Here, we use deep WGS data and multitissue RNA-seq expression data from 613 individuals in the Genotype-Tissue Expression (GTEx) project to comprehensively map SVs and to evaluate their impact on both common and rare gene expression changes in up to 48 tissue types (Supplemental Table S1). This study expands on our prior analysis of SV in 147 human samples from the GTEx cohort with RNA-seq expression data from 13 different tissues (Chiang et al. 2017) and is the most comprehensive study of SV-eQTLs to date. The expanded cohort size provides greater power to evaluate the impact and mechanisms of SV-associated gene expression changes, particularly for rare SVs.

## Results

### Variant calling

We mapped SVs in 613 individuals from the GTEx v7 release using LUMPY (Layer et al. 2014; Chiang et al. 2015), svtools (Larson et al. 2019), GenomeSTRiP (Handsaker et al. 2011, 2015), and the Mobile Element Locator Tool (MELT; see Methods) (Gardner et al. 2017). Variant calls were filtered and merged using the same approach as in our previous GTEx study (Chiang et al. 2017; Li et al. 2017), resulting in a total of 61,668 “high-confidence” SVs that are the basis for all subsequent analyses (Table 1). SNVs and small insertion deletion (indel) variants were mapped using GATK (McKenna et al. 2010) as part of the official v7 release from the GTEx Consortium.

**Table 1.**
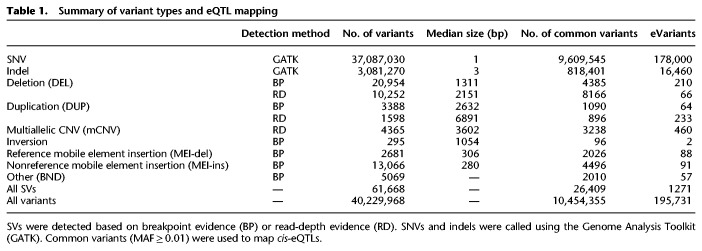
Summary of variant types and eQTL mapping

### Effects of common SVs

**Figure 1.**
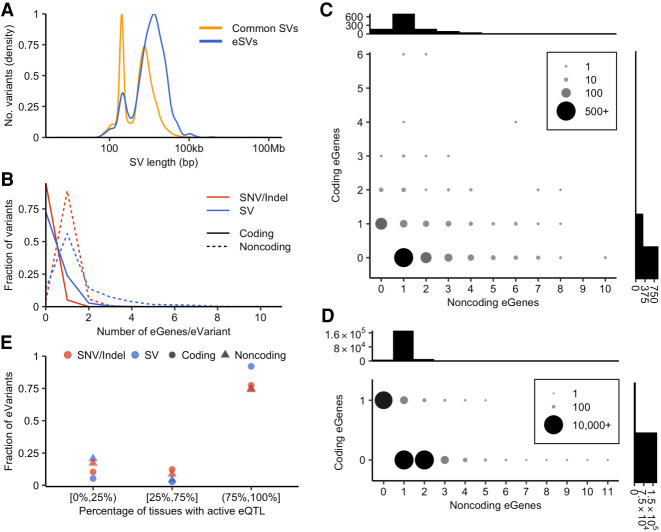
Features of SV-eQTLs. (*A*) Size distribution of eSVs compared with all common SVs. (*B*) Distribution of the number of eGenes per eVariant for SVs compared with SNVs and indels. “Coding” eGenes refer to eGenes whose exons are intersected by the associated eVariant, and “noncoding” eGenes are not intersected by the associated eVariant. Counts are shown for every eVariant; thus, eVariants with zero coding or zero noncoding eGenes are included in the distributions. (*C*,*D*) The number of eVariants, as shown by dot size and color, with the indicated combination of coding and noncoding eGenes, as defined above. Shown for SVs (*C*) and SNV/indels (*D*), with histograms showing the total number of eVariants, with the indicated number of associated coding or noncoding eGenes above the *y*- and *x*-axes, respectively. (*E*) Distribution of tissue specificity of eQTLs across tissues as evaluated by METASOFT, separated into the lowest quartile, middle two quartiles, and top quartile, for eQTLs in which the activity status is known in at least 43 of 48 evaluated tissues. The points indicate the fraction of SV-eQTLs or SNV- and indel-eQTLs that are active (m > 0.9) in the proportion of tissues indicated on the *x*-axis.

We performed *cis*-eQTL mapping of common variants (MAF ≥ 0.01) using a permutation-based mapping approach with FastQTL (Ongen et al. 2016), limiting comparisons to variants within 1 Mb of the transcription start site (TSS) of each gene. We performed eQTL analyses in each of the 48 tissues for which expression data were available for at least 70 individuals (Supplemental Table S1), and defined an eQTL as an eVariant/eGene pair detected in a given tissue. We performed a “joint” eQTL mapping analysis in which SVs, SNVs, and indels were simultaneously queried for eQTL status, allowing for direct comparisons between their properties and identification of a likely causal variant. An SV was the lead marker in 2.66% (7960/299,187) of eQTLs (Supplemental Table S2), although this is likely an underestimate of SV causality owing to inferior genotyping accuracy for SVs, which biases eQTL fine-mapping analyses against SVs. Although this estimate of the contribution of SVs is relatively small, it represents a 10.5-fold enrichment over the abundance of SVs in the genome. This result is consistent with our prior analysis of the initial 147 individuals from the GTEx cohort (Chiang et al. 2017). In the same 13 tissues evaluated in this previous study, the increased sample size used here allowed us to identify 617 genes with SV-eQTLs that were not identified in the smaller study, although 57 genes from the initial study are no longer SV-eQTLs. As expected, many eSVs are large, although we observe smaller eSVs as well (Fig. 1A). Furthermore, 71.82% (5717/7960) of all SV-eQTLs identified in this study are noncoding (Supplemental Fig. S1), meaning the SV does not intersect with any exons of its associated eGene. This figure is even more striking when eQTLs are collapsed across tissues, where 1907/2318 (82.27%) of unique eGene/eSV pairs are noncoding. This also suggests that coding SV-eQTLs are more constitutive as more of them are identified in multiple tissues.

A novel aspect of this study is that we used MELT to sensitively map MEI variants, including nonreference insertions that were not detected in our prior GTEx study. It has been proposed that MEIs may have broad effects on gene expression owing to their ability to disrupt genes, promote epigenetic gene silencing, and serve as alternate promoters (Chuong et al. 2017; Payer and Burns 2019); however, there has been scant data in humans to address these hypotheses. We found that only 0.12% (353/299,187) of eQTLs had an MEI as the lead marker. Although this is a 1.9-fold enrichment of predicted causal MEIs relative to their abundance (0.06% of common variants), MEIs were far less likely than other SV types to be the lead marker (e.g., mCNVs are enriched 45-fold, duplications 38-fold, and deletions 3.3-fold). It is unlikely that this relative depletion results simply from the small size of MEIs, as a size-matched analysis of MEIs and LUMPY deletions showed that only 2.74% of MEIs are eQTLs compared with 3.5% of deletions. When including LUMPY deletions of all sizes, 4.8% of deletions are eQTLs. This result is also unlikely to be explained by poor sensitivity or genotyping accuracy at MEIs considering that we detected slightly more MEIs per genome than a recent comprehensive long-read read study (Ebert et al. 2021)—with a mean of 1961 MEIs per genome versus 1637—and the linkage disequilibrium (LD) patterns at MEIs relative to nearby SNVs mirror that of LUMPY deletions (Supplemental Note; Supplemental Fig. S2). Thus, despite compelling molecular evidence for the functional potential of MEIs, our results suggest that they are only slightly enriched as causal eQTL variants relative to SNVs and indels and are depleted relative to other SVs, on average.

We found that not only do SVs have larger effect sizes compared to SNPs and indels, as noted in previous studies (Supplemental Fig. S1; Jakubosky et al. 2020; Chiang et al. 2017), they are also more likely to alter the expression of multiple nearby genes. Each eSV affects an average of 1.82 unique eGenes, whereas SNVs and indels affect an average of 1.09 unique eGenes. Although this effect is partially explained by large SVs that alter the copy number of multiple adjacent genes, there is also a significant difference for genes affected by noncoding eVariants: On average, eSVs affect 1.50 unique eGenes for which they do not intersect any exons of the eGene compared with an average of 1.04 unique eGenes for SNVs and indels (*P* = 1.02 × 10^−55^, one-sided Mann–Whitney *U* test) (Fig. 1B–D). These noncoding effects are most pronounced for duplications (*P* = 6.10 × 10^−53^) and mCNVs (*P* = 4.75 × 10^−56^), which are the only two categories of noncoding SVs that affect significantly more eGenes than point variants. This result indicates that causal SVs are generally more impactful than causal point variants, in terms of both their per-gene effect sizes and their potential to affect multiple genes. These results also suggest that SVs are more likely to disrupt key regulatory elements and/or alter higher-order genome architecture, allowing individual variants to affect multiple genes.

To investigate the functional mechanisms of expression-altering SVs, we defined a set of putative causal SVs using a score generated by taking the product of the causal probability calculated using CAVIAR (Hormozdiari et al. 2014) and the fraction of heritability attributed to the SV calculated using GCTA (Supplemental Table S3; Yang et al. 2011), as described previously (Chiang et al. 2017). At each eGene, we selected the SV within the *cis*-region that had the strongest association with the eGene's expression and allocated these 10,911 unique SVs into six bins on the basis of causality score quantiles, with the least-causal bin containing the 50% of SVs with the lowest scores. Next, we measured the enrichment of SVs in each causality bin at a diverse set of genomic annotations and in the core 15 chromatin segmentation states from the Roadmap Epigenomics Project using a permutation test based on shuffled genomic positions (see Methods) (Supplemental Figs. S3, S4). SVs in the most causal quantiles were strongly enriched in the exons of their associated eGenes, which is expected and confirms that our causality score is informative. We also observed an enrichment of causal SVs in the 10-kb regions upstream of the TSS and downstream from the 3′ UTR of the associated eGene. Additionally, there is a small enrichment of the causal SVs in segmental duplications, which is likely driven by large mCNVs at multicopy genes. However, predicted causal SVs were not enriched in any other genomic features tested, which suggests that although eSVs are generally found relatively close to their eGenes, they may be altering expression through diverse mechanisms, and our study is underpowered to identify enrichments in specific regulatory element classes. Alternatively, existing annotations may be insufficiently informative to detect functional enrichments for the variants and tissues analyzed here.

The number and diversity of tissues with available expression data allow us to evaluate the tissue specificity of eQTLs. We hypothesized that SVs might have more ubiquitous effects on gene expression than point variants owing to constitutively acting dosage changes or owing to complete deletion or duplication of regulatory elements rather than more subtle effects, for example, on transcription factor binding. To allow for facile comparisons between variant types, we limited this analysis to variant-gene pairs with a significant association in our eQTL analysis for which expression data were available across all 48 tissues. We used METASOFT (Han and Eskin 2011) to evaluate eQTL activity across all tissues and limited this analysis to eQTLs for which active (*m* > 0.9) or inactive (*m* < 0.1) status could be determined in at least 43 tissues. We found that coding SV-eQTLs are more constitutive than other eQTL classes, showing activity across a larger proportion of tissues compared with SNV- and indel-eQTLs (Fig. 1E). Whereas 92.16% of coding SV-eQTLs are constitutively active—defined here as active in >75% of tissues with known status—only 74.12% of coding SNV- and indel-QTLs are constitutive. However, the result at noncoding eQTLs is less clear: 74.86% of noncoding SV-eQTLs are constitutively active as defined above, and 74.12% of noncoding SNV- and indel-eQTLs are constitutive, which suggests that there are not significant differences between these variant categories. However, when we examine noncoding eQTLs that are active in 100% of tissues with known activity, 44.44% of noncoding SV-eQTLs are active in all known tissues compared with 26.23% of noncoding SNV- and indel-eQTLs (Supplemental Fig. S5). Overall, this analysis shows that coding SVs typically impact expression across many tissues, whereas smaller and noncoding variants tend to affect gene expression on a more tissue-specific basis. In contrast to coding SV-eQTLs, noncoding SV-eQTLs show similar patterns of tissue specificity to noncoding SNV- and indel-eQTLs, indicating that these variant types are likely to function through similar mechanisms. However, it is important to note that noncoding SV-eQTL activity could not be determined by METASOFT in many tissues (Supplemental Fig. S6), so it is possible that the true tissue specificity of noncoding SVs may differ from noncoding SNVs and indels. This appears to be the result of relatively large effect-size standard errors for SV-eQTLs that result from genotyping inaccuracies. Although METASOFT can determine cross-tissue eQTL activity when effect sizes are large despite large standard errors, as seen in coding SV-eQTLs, when effect sizes are small but effect size errors are large, the algorithm often cannot confidently judge activity (Supplemental Fig. S7).

### Effects of rare SVs

Rare SVs are enriched near genes with highly aberrant expression (Chiang et al. 2017) and are more likely to have large effect sizes compared with other variant types (Li et al. 2017). To assess the effects of rare SVs on gene expression, we identified genes in which individuals displayed highly aberrant gene expression levels compared with the data set as a whole. We limited this analysis to the 513 individuals of European descent to reduce the effects of population stratification and limited our analyses to the 47 tissues for which data were available for at least 70 European individuals (Supplemental Table S1). We defined 26,289 autosomal multitissue gene expression outliers (median |Z| ≥ 2 across all tissues in an individual) and 173,061 autosomal “tissue-restricted” outliers with highly aberrant expression (|Z| ≥ 4) in two or more tissues in the same individual. Next, we identified 13,768 “singleton” SVs no larger than 1 Mb in size that were positively genotyped in one individual. These rare SVs are strongly enriched within the gene body and flanking sequence of multitissue gene expression outliers compared with the null expectation in 1000 random permutations of the outlier sample names, with enrichment decreasing as flanking distance increases (Supplemental Fig. S8). The enrichment of rare SVs in close proximity (14.1-fold enriched within 5 kb; 95% confidence interval [CI], 8.7–25.1; *P* < 0.001) to multitissue gene expression outliers is consistent with our prior work (Chiang et al. 2017), but the increased power in this study allows us to observe enrichment at greater distances as well. At flanking distances as large as 50 kb, we observe a 6.4-fold enrichment (95% CI 4.9–8.8; *P* < 0.001) of rare SVs around multitissue outliers, suggesting that rare SVs contribute to rare expression differences even from relatively large genomic distances. Importantly, because gene expression values can only decrease to zero, a conservative *Z*-score limit such as the one used for tissue-restricted outliers favors gene expression outliers with increased expression, thus limiting our ability to detect SVs associated with decreased expression (Supplemental Fig. S9). However, these conservative outlier definitions, combined with the above enrichment results, provide confidence in the set of outlier-associated SVs.

**Figure 2.**
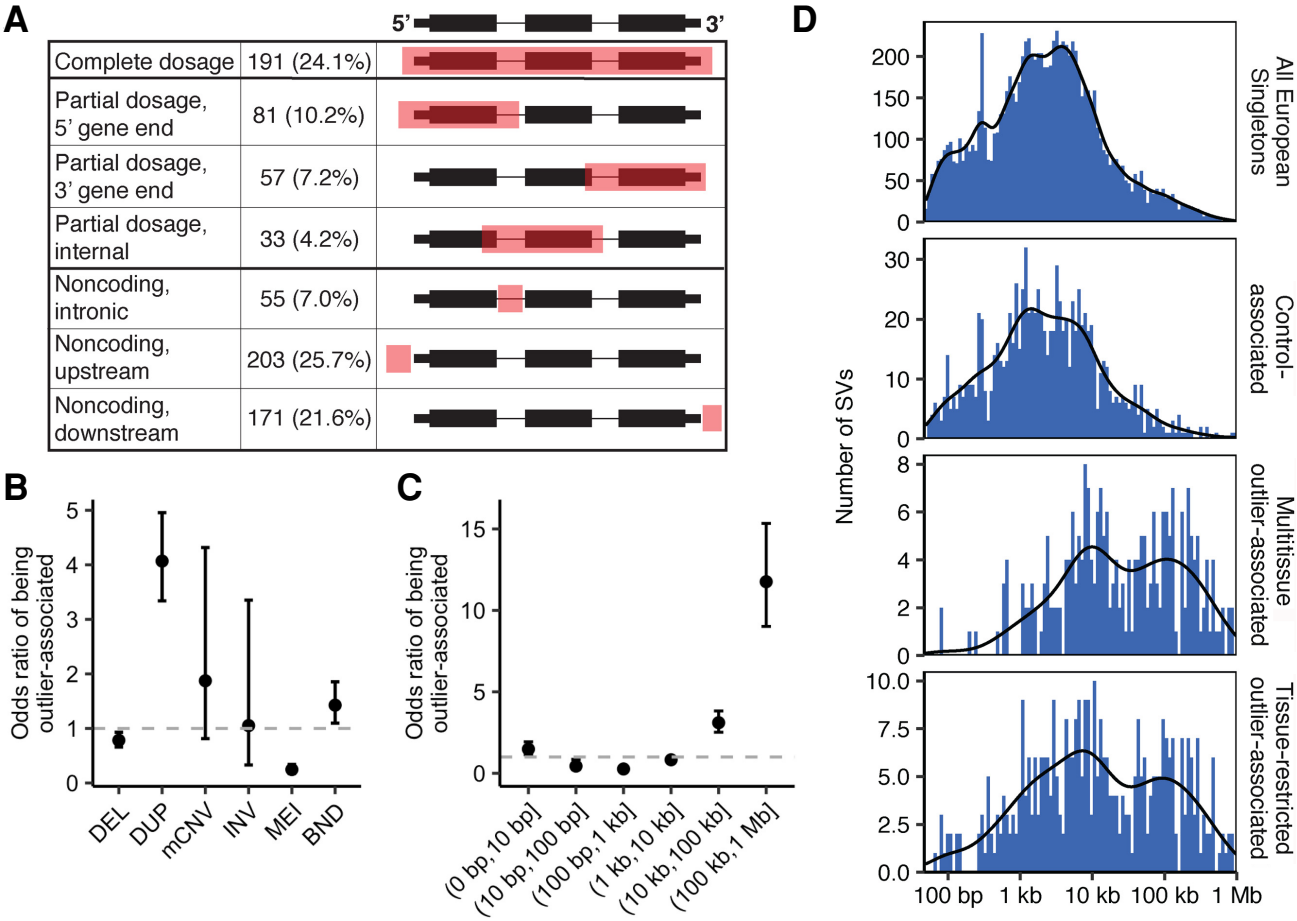
Features of outlier-associated SVs. (*A*) Location of outlier-associated SVs relative to their associated outlier gene and the number of SV/outlier gene associations identified in each category. Percentages indicate the fraction of outlier/SV pairs found at each relative location compared with the total number of SV/outlier gene associations. Note that this definition allows one SV to be associated with multiple outlier genes, and thus, the SV is counted in multiple categories. Gene diagrams provide examples of possible SV location, shown in red, relative to the outlier gene. (*B*,*C*) Odds ratio (OR) of being outlier-associated by SV type (*B*) and SV size (*C*) for the SV category of interest compared with all other SVs. Note that BNDs were excluded from the size OR calculations owing to their ambiguous nature and thus size. (*D*) Distribution of SV sizes for singleton SVs <1 Mb identified in European individuals that were used in outlier analyses. Panels depict size distributions for all European-cohort singletons, control-associated singletons, multitissue outlier-associated singletons, and tissue-restricted outlier-associated singletons.

A total of 539 unique outlier-associated SVs are located in the gene body and 50-kb flanking region of gene expression outliers (Fig. 2A; Supplemental Table S4). A majority of these (62.34%; 336/539) are noncoding SVs that do not affect the coding sequence of one or more expression outliers. This contradicts the general assumption that rare SVs typically act through gene dosage effects. In total, 16.92% (31,978/188,988) of expression outliers are associated with a rare SV, although outliers can also arise via nongenetic mechanisms. To evaluate the relative potential of different SV types or sizes to cause expression outliers, we calculated the odds ratio (OR) of being outlier-associated for the SV category of interest compared with all other SVs. Duplications (OR 4.07) and mCNVs (OR 1.87) are most likely to be associated with an expression outlier, MEIs are least likely (OR 0.25) (Fig. 2B), and larger SVs are more likely to be outlier-associated regardless of type (Fig. 2C). However, many outlier-associated SVs are smaller in size (Fig. 2D). For example, 13.33% (50/375) of SVs associated with tissue-restricted outliers are <1 kb, and nearly half (49.33%; 185/375) are <10 kb. Multitissue outlier-associated SVs tend to be slightly larger, with only 4.98% (12/241) <1 kb and 35.27% (85/241) <10 kb. These results provide further evidence that rare SVs often affect gene expression through more complex mechanisms than large, dosage-altering events.

**Figure 3.**
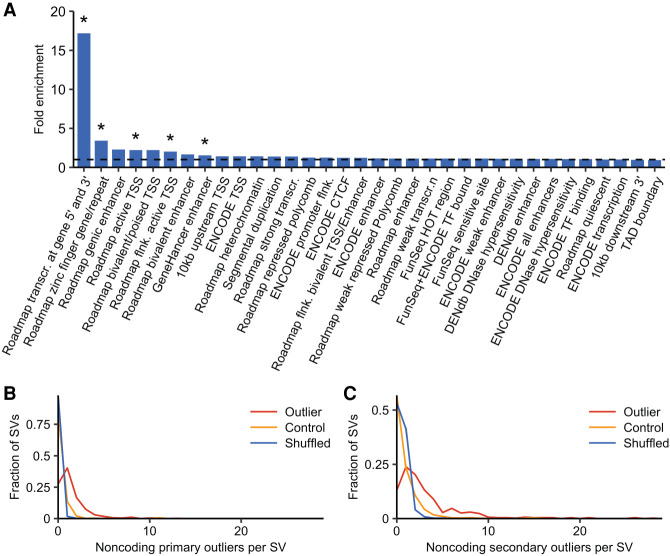
Mechanistic insights into outlier-associated SVs. (*A*) Enrichment of outlier-associated SVs in functional genomic annotations compared with control-associated SVs. Asterisks indicate statistical significance based on a Fisher's exact test with Bonferroni correction for multiple testing. (*B*,*C*) The distribution of the number of noncoding primary (*B*) and secondary (*C*) outliers found within 1 Mb of the region surrounding tissue-restricted outlier-associated SVs, control-associated SVs, and a shuffled null.

We next sought to determine if rare outlier-associated SVs are enriched in annotated genomic features. Although there was little signal in our enrichment analysis of common SVs, as described above, rare variants typically have larger effect sizes and are more likely to be deleterious. For this analysis, we defined a set of “control” SVs that are located within or near genes but do not show expression effects. We identified 1405 singleton SVs (1327 noncoding) located within 50 kb of autosomal genes that showed consistent expression levels (|Z| < 1) across all tissues in an individual. Although this is not an ideal set of control SVs considering that some may in fact alter gene expression in tissues or at developmental time points for which expression was not measured, it is nonetheless a relatively conservative set of likely-nonfunctional SVs that can be used for comparison to outlier-associated SVs. We examined the overlap of both outlier- and control-associated noncoding SVs with annotated genomic features and with segmentation states from the Roadmap Epigenomics Project core 15-state model (Fig. 3A). We observed significant enrichment of outlier-associated SVs in 5 of the 34 evaluated features and chromatin states (Fisher's exact test; Bonferroni *P* < 0.05). Most of these significant associations are in Roadmap Epigenomics Project segmentation states in close proximity to transcribed genes, including transcription at the 5′ and 3′ end of genes showing both promoter and enhancer signatures, active TSSs, and regions flanking active TSSs. We also observed significant enrichment in the Roadmap Epigenomics Project segmentation state associated with zinc finger protein genes and in enhancer annotations from GeneHancer. It is important to note, however, that the number of overlaps observed in this analysis is small and that increased power might change these results. Thus, although rare SVs appear to have large effects on gene expression, most existing functional annotations are not very informative. Consistent with this, the distribution of SV impact scores (Ganel et al. 2017) is not significantly different between expression-altering SVs and control SVs (Supplemental Fig. S10).

We found that 115 (21.34%) outlier-associated SVs are associated with more than one expression outlier and that eight (1.48%) are associated with five to nine expression outliers, suggesting that many rare SVs may have regional effects. To evaluate these broader regional effects of rare expression-altering SVs, we relaxed the definition for aberrant expression to generate a set of “secondary” expression outliers in which the tissue-restricted (“primary”) outlier absolute *Z*-score cutoff was reduced to three in at least two tissues. We found significantly more primary and secondary outliers within 1 Mb of the 469 tissue-restricted outlier-associated SVs compared with the 1405 control-associated SVs and with a null distribution in which we randomly shuffled the sample names of outlier-associated SVs 1000 times and calculated the median number of associated outlier genes (Fig. 3B,C). This increase is especially pronounced for secondary outliers whose coding regions do not overlap with the associated SV. We observe that noncoding outlier-associated SVs are associated with an average of 1.44 primary outliers (|Z| ≥ 4) compared with an average of 0.02 associated primary outliers surrounding the shuffled null SVs (*P*-value = 2.78 × 10^−106^; one-sided Mann–Whitney *U* test). These differences remain for secondary outliers, with an average of 3.34 secondary outliers found in the expanded region surrounding noncoding outlier-associated SVs compared with an average of 0.54 secondary outliers for the shuffled null (*P*-value = 4.94 × 10^−76^; one-sided Mann–Whitney *U* test). These results suggest that rare SVs have far-reaching effects on gene expression and that these effects are primarily driven by noncoding regulatory mechanisms rather than by changes to gene copy number.

## Discussion

We have comprehensively mapped SVs from WGS data in 613 individuals from the GTEx data set and analyzed the impact of both common and rare SVs on human gene expression. Our findings confirm results from previous analyses that SVs make an outsized contribution to common gene expression changes compared with their abundance in the genome and play an important role in rare gene expression differences (Chiang et al. 2017). A novel aspect of this study is the inclusion of a comprehensive set of MEI insertions, including those present in the GTEx samples but not the reference genome. We observed that MEIs do not play an especially important role in determining gene expression differences. In contrast, we found that mCNVs play an extremely impactful role, being 45-fold enriched among eQTL lead markers compared with their abundance in the genome and being more likely to be associated with gene expression outliers (OR = 1.88). mCNVs were found to give rise to most human variation in gene dosage (Handsaker et al. 2015), but our findings indicate that noncoding functional mCNVs are also abundant in the human genome.

One of the major motivators for studies such as this one is to understand the role of genetic variation in affecting gene transcription. Expression-altering SVs were not well correlated with any specific functional annotations other than proximity to genes, and thus, existing annotations are unlikely to be informative for modeling functional variant effects. This may simply be owing to a lack of power given that SVs are such a diverse class of variants that can affect large genomic segments and have the potential to affect gene expression through diverse mechanisms, and our sample size is limited to 11,026 common SVs and 539 rare SVs predicted to be functional. Alternatively, the annotations currently available may be inadequate.

Nonetheless, it is clear that SVs have broad regional impacts on human gene expression, with individual variants frequently affecting multiple genes. These effects are not driven by large CNVs that alter the dosage of multiple coding sequences, as one might naively expect, but are most commonly observed for noncoding variants: Common noncoding eSVs affect an average of 1.50 unique genes, and rare noncoding SVs are associated with an average of 1.44 primary expression outlier genes. This observation suggests a mechanism by which rare noncoding SVs may be especially deleterious, and may help explain why prior work has estimated that a large number of rare noncoding deletions—an average of 19.1 per individual—appear to be under strong purifying selection (Abel et al. 2020). Furthermore, the burden of de novo CNVs has been associated with autism spectrum disorder, including for noncoding variants (Turner et al. 2017; Turner and Eichler 2019). Our results provide a mechanism through which individual noncoding SVs can have strong and potentially pleiotropic effects and thus a higher potential to contribute to disease.

Although this study represents the most comprehensive analysis of the impact of SVs on human gene expression to date, our call set is missing some of the most repetitive classes of SV, such as short tandem repeats. As long-read sequencing and variant calling methods improve, we will be able to gain additional insights into repetitive variants in the most complex regions of the genome. Despite the limitations of short-read sequencing data, this study shows the importance of comprehensive variant detection when evaluating genomic variants that contribute to gene expression and disease. SVs have a disproportionately large effect on common and rare gene expression changes and often affect multiple genes. Our findings reinforce the importance of comprehensive variant detection in the design of future trait mapping studies.

## Methods

### Call set generation

We obtained 613 WGS BAM files from the GTEx v7 release (NCBI database of Genotypes and Phenotypes [dbGaP; https://www.ncbi.nlm.nih.gov/gap/] accession phs000424.v7.p2, accessed June 1, 2016). These data were aligned to GRCh37, and we did not realign to GRCh38 for this analysis to allow for comparison between SVs and the SNV and indel data available for this GTEx release. Our use of GRCh37 rather than the newer GRCh38 will not significantly affect the results or conclusions of this study, considering that the vast majority of loci have similar sequence content and structure; however, we note that CNV calls at specific structurally complex loci can vary somewhat between references. SV calls were generated using both the SpeedSeq v0.1.1 pipeline (Chiang et al. 2015), which performs sample-level breakpoint detection via LUMPY v.0.2.13 (Layer et al. 2014) followed by population-scale merging and genotyping of SV calls via svtools v0.3.1 (Larson et al. 2019), and the GenomeSTRiP v2.00.1636 read-depth analysis pipeline (Handsaker et al. 2011), as described in our preliminary GTEx study (Chiang et al. 2017). The GenomeSTRiP false-discovery rate (FDR) was evaluated based on available Illumina Human Omni 5M gene expression array data (n = 161) using the GenomeSTRiP IntensityRankSumAnnotator. We limited GSCNQUAL to one or more for GenomeSTRiP deletions and to eight or more for multiallelic CNVs, corresponding to an FDR of 10%. The GSCNQUAL cutoff for GenomeSTRiP duplications was set at 17 or more, the point at which the FDR plateaued at 15.1% and did not fluctuate more than ±1% for over 50 steps of increasing GSCNQUAL score. Redundant LUMPY and GenomeSTRiP calls were merged as previously described (Chiang et al. 2017). Additionally, we ran MELT v2.1.4 using MELT-SPLIT to identify *Alu*, SVA, and LINE-1 insertions into the test genomes (Gardner et al. 2017). We retained MELT calls categorized as “PASS” in the VCF info field that had an ASSESS score of three or more and SR count of three or more. Genome Analysis Toolkit (GATK) HaplotypeCaller v3.4 (McKenna et al. 2010) SNV and indel calls were obtained from the GTEx Consortium (dbGaP accession phs000424.v7.p2, accessed June 1, 2016). We use allele balance instead of genotype for the analyses described in this paper because it is tolerant to alignment inefficiencies for the alternate SV allele. For MEIs identified by MELT, we converted generated genotypes (0/0, 0/1, 1/1) to integer values (0, 1, 2) that were used as a proxy for allele balance to allow for comparable analyses on these variants.

### Common eQTL mapping

We mapped *cis-*eQTLs in each of the 48 tissues for which both WGS data and RNA-seq data were available in 70 or more individuals. Available tissues and those used in each analysis are listed in Supplemental Table S1. We refer to EBV-transformed lymphocytes and transformed fibroblasts as tissue types throughout this study for convenience. Biospecimen collection, RNA-seq data alignment, RPKM calculations, and data normalization were previously described (Lappalainen et al. 2013; Chiang et al. 2017).

We selected common genetic markers, defined as having MAF ≥ 0.01, for eQTL mapping. We performed a joint *cis*-eQTL analysis that included 26,409 common SVs, as well as 9,609,545 common SNVs and 818,401 common indels detected using GATK, to allow for a fair comparison of the contribution of different variant types. We used FastQTL v2.184 (Ongen et al. 2016) to perform *cis*-eQTL mapping, customized to accommodate the unique architecture of SVs (Chiang et al. 2017), using a *cis* window of 1 Mb on either side of the TSSs of autosomal and X Chromosome genes with a permutation analysis to identify the most significant marker for each gene. For each tissue, we applied the same covariates described by Chiang et al. (2017). We corrected for multiple testing at the gene level using the Benjamini–Hochberg method with a 10% FDR.

To evaluate the quality of our MEI-eQTLs, we performed a size-matched analysis by randomly selecting 286 LUMPY deletions to match the size distribution, measured in 50-bp bins, of the 6458 MEIs included in this study. We then calculated the percentage of selected LUMPY deletions that cause an eQTL compared with the number of MEI-eQTLs. We only used deletions detected by LUMPY for this analysis because they are mapped to high resolution and because we can be confident of their size. However, there are few deletions as small as MEIs, and thus, only a small subset of deletions was selected in order to match the two size distributions.

We calculated R^2^ between each SV and its best tagging SNV using the GenomeSTRiP TagVariantsAnnotator (Handsaker et al. 2015).

### Feature enrichment

To evaluate whether SVs that cause common gene expression changes are enriched in particular genomic features, we calculated a previously described causality score (Chiang et al. 2017) generated by taking the product of the SV heritability fraction obtained from GCTA (Yang et al. 2011) and the causal probability generated by CAVIAR (Hormozdiari et al. 2014) for the strongest-associated SV within the *cis* region of each eGene. No associated SVs were identified in 199 eQTLs owing to the subset of samples with available data in the relevant tissue and thus were not included in enrichment analyses. GCTA heritability estimates could not be calculated for a small number of eQTLs (6146/299,187) owing to nonpositive definite matrices, likely resulting from small sample sizes, and these loci were excluded from feature enrichment analyses. For SVs that were associated with multiple eQTLs or the same eQTL in multiple tissues, we selected the eQTL (tissue/gene pair) for which the SV had the highest causality score. SVs were allocated into bins based on causality score quantiles, with the first bin consisting of SVs in the bottom 50% of causality scores and the other five consisting of deciles of the top 50% of scores.

Next, we counted the number of SVs in each bin that intersected with various genomic annotations. We allowed 1 kb of flanking distance surrounding all annotations with the following exceptions: GENCODE exons, no flanking distance; proximity to TSS and 3′ gene end, 10 kb of directional flanking distance; topologically associated domain boundaries, 5 kb of flaking distance; and Roadmap Epigenomics segmentation states, no flanking distance. SVs associated with multiple eGenes were considered to touch an eGene if they overlapped with the exons of any associated gene. SVs that touched an exon of an associated eGene were excluded from all feature enrichment analyses except for the enrichment of affected eGene exons. To generate a shuffled null for comparison, SVs within each causality bin were shuffled with BEDTools v2.23.0 (Quinlan and Hall 2010) into nongapped regions of the genome within 1 Mb of the TSS of a gene. We did not allow shuffled SVs to intersect any exons of their new eGene for all feature enrichment analyses, except for the enrichment of affected eGene exons. We calculated the fold enrichment of the number of SVs that intersect with each genomic feature compared with the median number of intersections observed for 100 randomly shuffled sets within each causality bin. These shuffled sets were also used to empirically derive the 95% confidence intervals.

The flanking distances indicated above were included to be consistent with our prior publication (Chiang et al. 2017), in which we observed that the enrichments were notably stronger for certain annotations with imprecise boundaries when we included the padding. However, we repeated the enrichment analysis for this study without the padding approach and found that the results look very similar to the results with padding and do not alter our conclusions (Supplemental Fig. S11).

Regions 10 kb upstream of TSSs and downstream from 3′ gene ends were defined based on GENCODE v19 gene positions. DNase I hypersensitive regions and enhancer regions with a minimum support of two were obtained from the Dragon ENhancers database (DENdb) (Ashoor et al. 2015). We downloaded FunSeq 2.1.0 (Fu et al. 2014) regions and topologically associated domain boundaries from human embryonic stem cells from investigator websites (http://archive.gersteinlab.org/funseq2.1.0_data/ and http://compbio.med.harvard.edu/modencode/webpage/hic/hESC_domains_hg19.bed). GeneHancer (Fishilevich et al. 2017) enhancer regions for b38 were downloaded from the UCSC Genome Browser (Kent et al. 2002) and lifted over to b37 using CrossMap v0.2.6 (Zhao et al. 2014). Regions defined by the ENCODE Project (The ENCODE Project Consortium 2012) were downloaded from the UCSC Genome Browser. To evaluate the intersection with the chromatin segmentation state annotations from the Roadmap Epigenomics Project (Roadmap Epigenomics Consortium et al. 2015), we downloaded the core 15-state model annotations for all 127 available epigenomes (https://egg2.wustl.edu/roadmap/data/byFileType/chromhmmSegmentations/ChmmModels/coreMarks/jointModel/final). We used BEDTools multiIntersectBed (Quinlan and Hall 2010) to identify genomic intervals where each of the 15 annotations is found in at least 10 of the 127 available epigenomes, and we used these collapsed regions as the annotation intervals for SV intersections.

### eQTL tissue specificity

We selected significant gene-variant pairs identified in eQTL mapping with available expression data available across all 48 tissues in which the eQTL analyses were performed. These pairs were only required to have a significant eQTL in one tissue. We used METASOFT v2.0.0 (Han and Eskin 2011) to perform a meta-analysis of the selected eQTL effect sizes and their standard errors across all 48 tissues. METASOFT uses a mixed effects model (RE2) to generate a posterior probability that an effect exists in each tissue (*m*-value) (Han and Eskin 2012). To allow computational feasibility with the relatively large number of tissues sampled, the Markov chain Monte Carlo (MCMC) method was used to approximate these values. The *m*-values generated indicate whether a tested eQTL is active (*m* > 0.9), is inactive (*m* < 0.1), or has ambiguous activity (0.1 ≤ *m* ≤ 0.9). Only eQTLs with at least 43 tissues having known (active or inactive) activity were included in analyses. eQTLs with active status in at least 75% of tissues with known activity were defined as “constitutively active.”

### Identification of expression outliers

We limited outlier analyses to the 513 European individuals, the largest subpopulation in the cohort, who had available WGS data. We performed *Z*-transformation of PEER-corrected expression values without quantile normalization across the 47 tissues for which RNA-seq data were available from the GTEx Consortium for at least 70 European individuals (Supplemental Table S1). We defined two sets of gene expression outliers (gene/sample pairs) among these individuals: “multitissue” expression outliers in which an individual's absolute median *Z*-score of a gene's expression across all available tissues was two or more, as previously described (Chiang et al. 2017), and “tissue-restricted” outliers in which an individual's absolute *Z*-score for a gene's expression was four or more in at least two different tissues. The two-tissue requirement was necessary to eliminate false-positive expression outliers resulting from individual tissues with systematically aberrant gene expression profiles for an individual. Additionally, we defined a set of control gene/sample pairs in which an individual's absolute *Z*-score of a gene's expression was less than one across all tissues for which RNA-seq data were available. For all definitions, we limited to gene/sample pairs with data available in at least five tissues. We removed one individual (GTEX-14753) from this analysis owing to an excessive number of expression outliers.

### Rare variant association with expression outliers

We identified 13,769 SVs that were positively genotyped in no more than one individual in the European cohort. Because large rare SVs tend to affect gene expression through dosage changes, we removed 12 variants >1 Mb in size from this analysis. We calculated the enrichment of singleton SVs overlapping with multitissue outlier transcripts and the flanking 5-kb sequence by randomly shuffling the outlier individual names 1000 times to determine the median number of times a rare variant randomly co-occurred with an outlier, as described previously (Chiang et al. 2017). We also performed the reciprocal analysis, counting the number of outliers that co-occurred within 5 kb of a rare SV. We repeated these calculations for increased outlier-flanking regions of 10 kb, 25 kb, 50 kb, and 100 kb. We calculated the OR of being outlier-associated by dividing the ratio of outlier-associated SVs to non-outlier-associated SVs in a category of interest (SV type or size) by the ratio of outlier-associated SVs to non-outlier-associated SVs for all SVs not included in the category.

### Feature enrichment for outlier-associated SVs

We performed intersections between the 373 noncoding outlier-associated SVs and the same genomic features and chromatin segmentation states evaluated for eSVs. The above intersections were repeated for the 1327 noncoding control-associated SVs. We calculated the fold enrichment of outlier-associated SVs in each feature compared with control-associated SVs, and determined significant enrichments using a Fisher's exact test with Bonferroni correction for multiple testing.

### Regional effects of rare SVs

To evaluate the broader regional effects of rare, gene expression-altering SVs, we counted the number of tissue-restricted outlier genes, referred to as “primary” outliers, located in the spanning region and 1 Mb of flanking sequence both upstream of and downstream from the 469 SVs previously identified as being associated with a tissue-restricted expression outlier. We repeated this analysis with a relaxed definition of tissue-restricted expression outliers, referred to as “secondary” outliers, in which the absolute *Z*-score cutoff was reduced from |*Z*| ≥ 4 to |*Z*| ≥ 3. We compared the number of primary and secondary outliers found in the expanded region surrounding outlier-associated SVs to the expanded region surrounding the 1405 control-associated SVs. Finally, because the controls defined above do not represent a null expectation, we performed 1000 random permutations of the outlier-associated SV sample names and calculated the median number of associated primary and secondary outliers for each SV in order to determine how frequently rare expression-altering SVs co-occurred with primary and secondary outliers in random individuals.

## Data access

The SV genotype data generated in this study have been submitted to AnVIL and can be downloaded from the GTEx v7 workspace (https://app.terra.bio/#workspaces/anvil-datastorage/AnVIL_GTEx_V7_hg19). A VCF file containing all SV calls and genotypes, including non-PASS variants that failed quality filters (GTEx_v7.sv.low_pass.vcf), and an accompanying README file (GTEx_v7_SV_README.txt) are available in this workspace. A Terra account and dbGaP access to GTEx (accession number phs000424) are required.

## Supplementary Material

Supplemental Material
