# Supplementary material for: Structural variants are a major source of gene expression differences in humans and often affect multiple nearby genes
Source: Genome Res. 2021 Dec;31(12):2249–57. doi: 10.1101/gr.275488.121 (PMC8647827; doi:10.1101/gr.275488.121)
Supplement: Supplemental Material [file supp_31_12_2249__DC1.html]

Structural variants are a major source of gene expression differences in humans and often affect multiple nearby genes — Supplemental Material 

# Structural variants are a major source of gene expression differences in humans and often affect multiple nearby genes

## Supplemental Material

- Supplemental\_Table\_S1.xlsx
- Supplemental\_Table\_S2.xlsx
- Supplemental\_Table\_S3.xlsx
- Supplemental\_Table\_S4.xlsx
- Supplemental\_Material.pdf
