# Supplementary material for: Structural variants are a major source of gene expression differences in humans and often affect multiple nearby genes
Source: Genome Res. 2021 Dec;31(12):2249–57. doi: 10.1101/gr.275488.121 (PMC8647827; doi:10.1101/gr.275488.121)
Supplement: Supplemental Material [file supp_gr.275488.121_Supplemental_Material.pdf]

Supplemental Material

Structural variants are a major source of gene expression differences in humans and often affect multiple nearby genes

Alexandra J. Scott, Colby Chiang, Ira M. Hall

Supplemental Fig. S1: eQTL effect size distributions 2

Supplemental Note: Validation of MEI contribution to eQTLs 3

Supplemental Fig. S2: SV linkage to best tagging SNV 4

Supplemental Fig. S3: Enrichment of SV-eQTLs in annotated genomic features 5

Supplemental Fig. S4: Enrichment of SV-eQTLs in Roadmap epigenomic segmentation states 6

Supplemental Fig. S5: Distribution of eQTL tissue specificity across tissues 7

Supplemental Fig. S6: Distribution of eQTLs with unknown status 8

Supplemental Fig. S7: Distribution of eQTL effect sizes and effect size standard errors 9

Supplemental Fig. S8: Enrichment of singleton SVs near multi-tissue expression outliers 10

Supplemental Fig. S9: Distribution of gene expression outlier effect sizes 11

Supplemental Fig. S10: Distribution of outlier-associated SV impact scores 12

Supplemental Fig. S11: Enrichment of SV-eQTLs in annotated genomic features without padding 13

Supplemental Tables are separate from this file and can be downloaded individually.

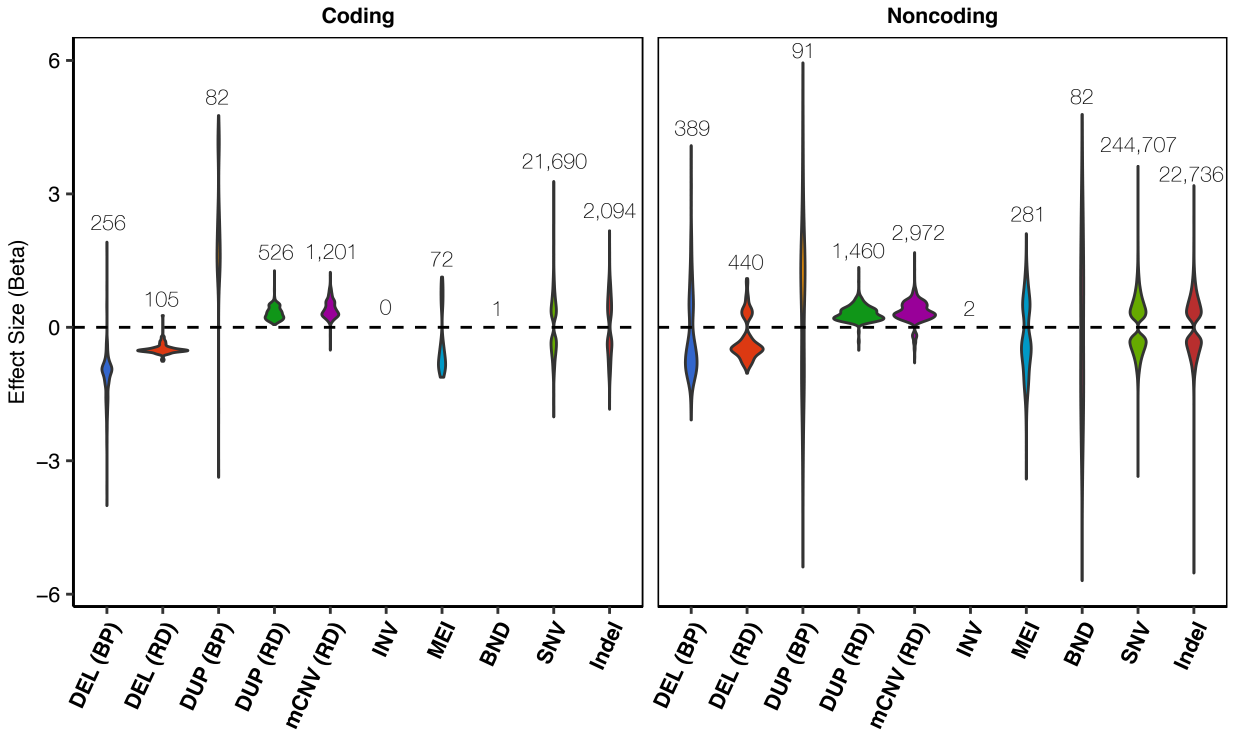

Supplemental Fig. S1. eQTL effect size distributions for coding and noncoding variants of each type with the number of eQTLs shown above the distribution. Deletions and duplications are separated by evidence used for variant discovery, either breakpoint (BP) or read-depth (RD).

Supplemental Note: Validation of MEI contribution to eQTLs

We quantified our MEI call set by comparing the number of MEIs mapped here to a call set from a recent study by the Human Genome Structural Variant Consortium (HGSVC) that was generated using long-read sequencing data (Ebert et al. 2021). We observed a mean of 1,961 MEIs per genome (median 1,528) while the HGSVC study mapped a mean of 1,637 MEIs per genome (median 1,258). Thus, it appears that we are detecting slightly more MEIs per genome despite our use of short-read WGS data, not fewer as one might naively expect. We believe that this is due to the fact that LUMPY and MELT are extremely good at detecting MEIs within relatively non-repetitive sequence, and because MEI detection is not trivial using long-read data, where the mapping methods are less mature. While there are some minor differences in how MEIs are classified based on annotations, overall, these data support the sensitivity of our MEI call set.

We also examined the linkage disequilibrium (LD) patterns at MEIs compared to other variant types by measuring R^2 between each SV and its most tightly linked SNV (**Supplemental Fig. S2**). The patterns of LD observed at MEIs closely mirrors the patterns observed at LUMPY deletions, and we know from extensive prior work that deletions are the easiest SV type to detect and genotype accurately. In contrast, other variant types such as tandem duplications and multi-allelic CNVs are not as well tagged due to inferior genotyping quality and recurrent mutation.

References

Ebert P, Audano PA, Zhu Q, Rodriguez-Martin B, Porubsky D, Bonder MJ, Sulovari A, Ebler J, Zhou W, Serra Mari R, et al. 2021. Haplotype-resolved diverse human genomes and integrated analysis of structural variation. Science 372. http://dx.doi.org/10.1126/science.abf7117.

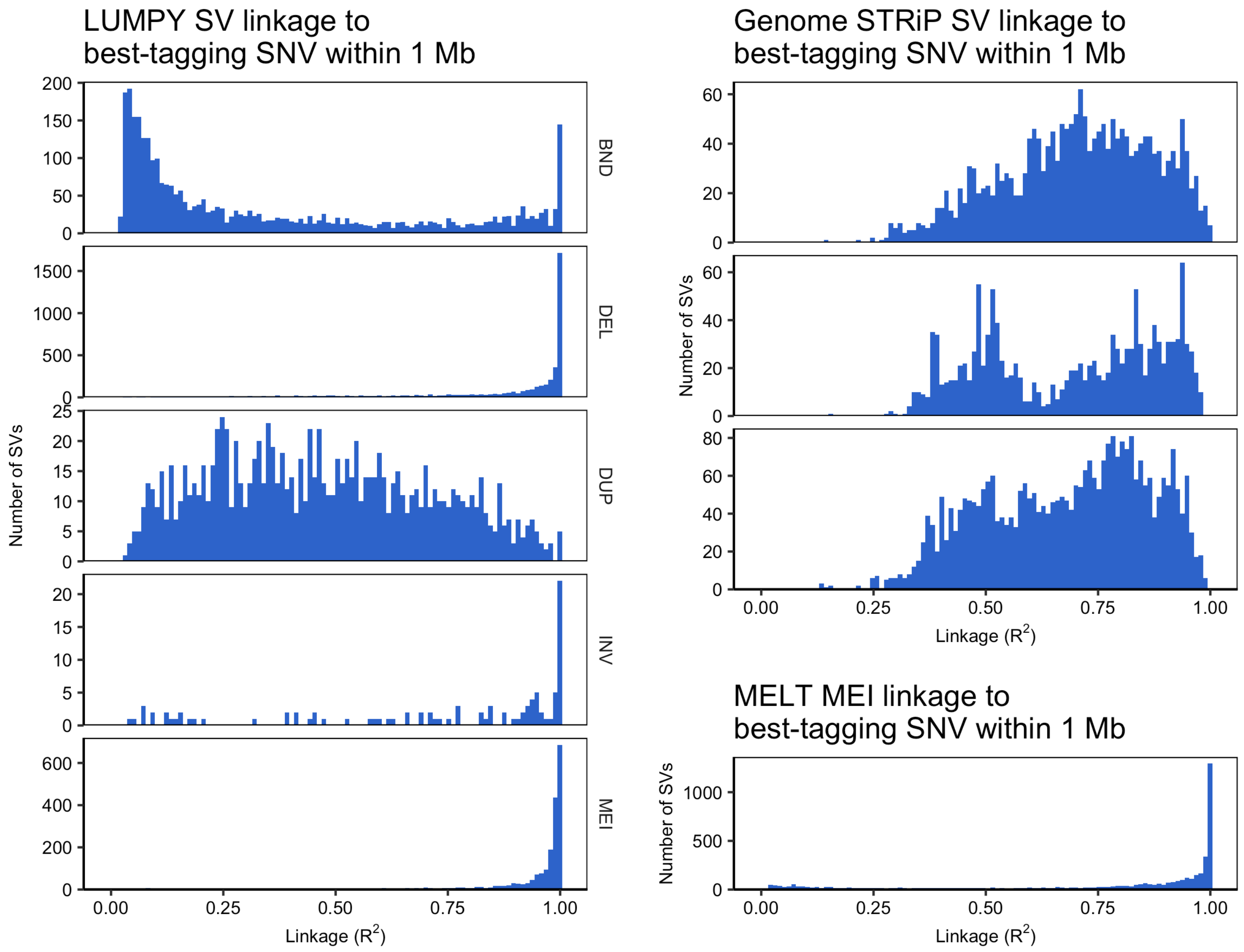

Supplemental Fig. S2. Distribution of linkage disequilibrium, measured by R^2, between SVs detected by LUMPY, Genome STRiP and MELT and the most tightly linked SNV to each SV.

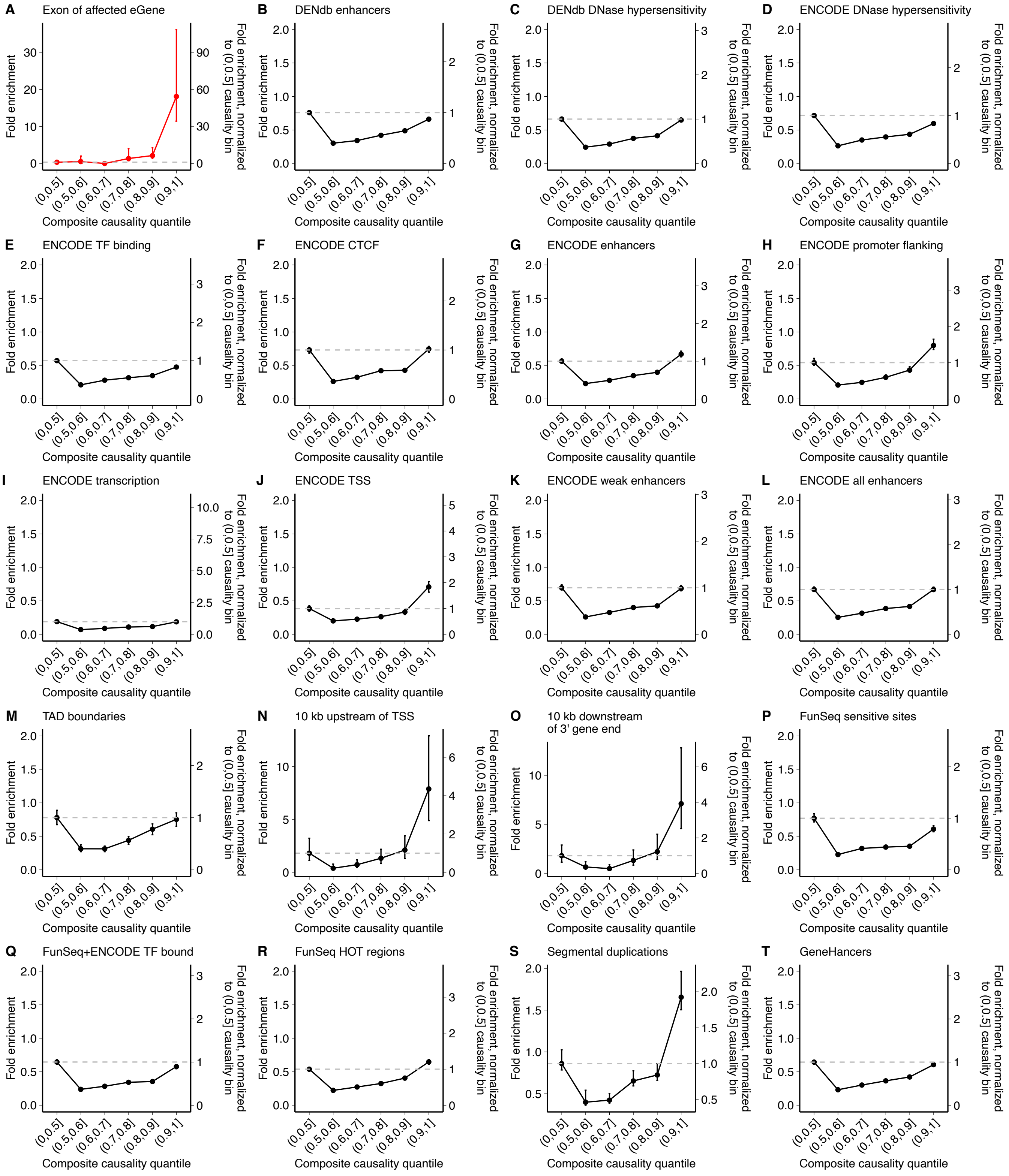

Supplemental Fig. S3. Feature enrichment of SV-eQTLs. Fold enrichment and 95% confidence intervals (based on 100 random shuffled sets of the positions of SVs in each bin) for the overlap between the most significant SV for each eGene and various annotated genomic features. (**A**) Enrichment of SVs in each causality bin for intersections with exons of the affected eGene. (**B-T**) For the remaining plots, SVs that overlapped with an exon of the affected eGenes were excluded. Enrichment was only observed in the 10-kb regions upstream (**N**) and downstream (**O**) of TSSs and in segmental duplications (**S**), which is consistent with the known concentration of SVs in architecturally complex genomic regions.

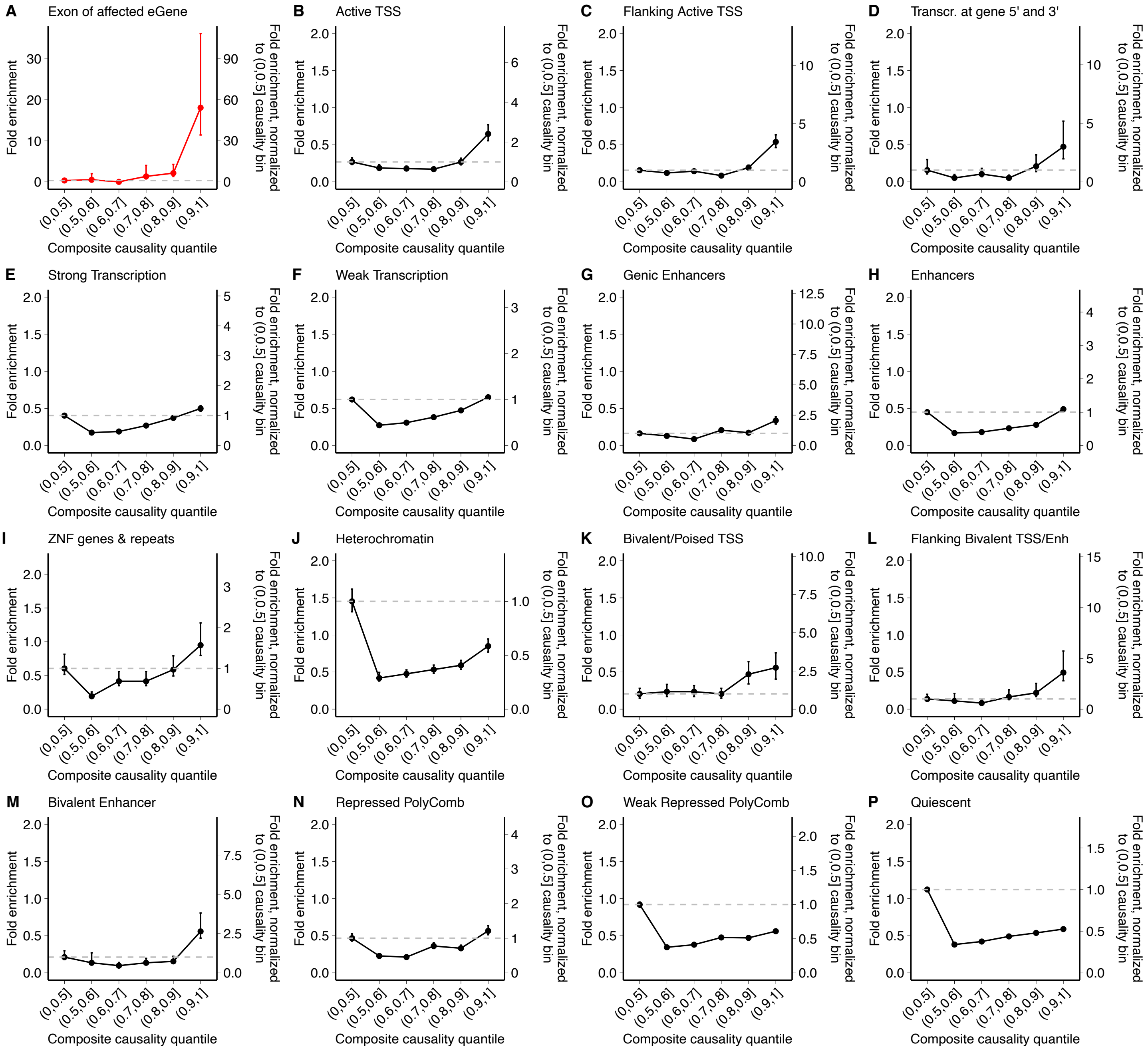

Supplemental Fig. S4. Enrichment of SV-eQTLs in Roadmap Epigenomics segmentation states. Fold enrichment and 95% confidence intervals (based on 100 random shuffled sets of the positions of SVs in each bin) for the overlap between the most significant SV for each eGene and various annotated genomic states. (**A**) Enrichment of SVs in each causality bin for intersections with exons of the affected eGene. (**B-P**) For the remaining plots, SVs that overlapped with an exon of the affected eGenes were excluded. We identified genomic intervals where each of the 15 Roadmap Epigenomics segmentation states are found in at least 10 of the 127 available epigenomes and identified SVs in each causality bin that overlapped with these collapsed genomic intervals.

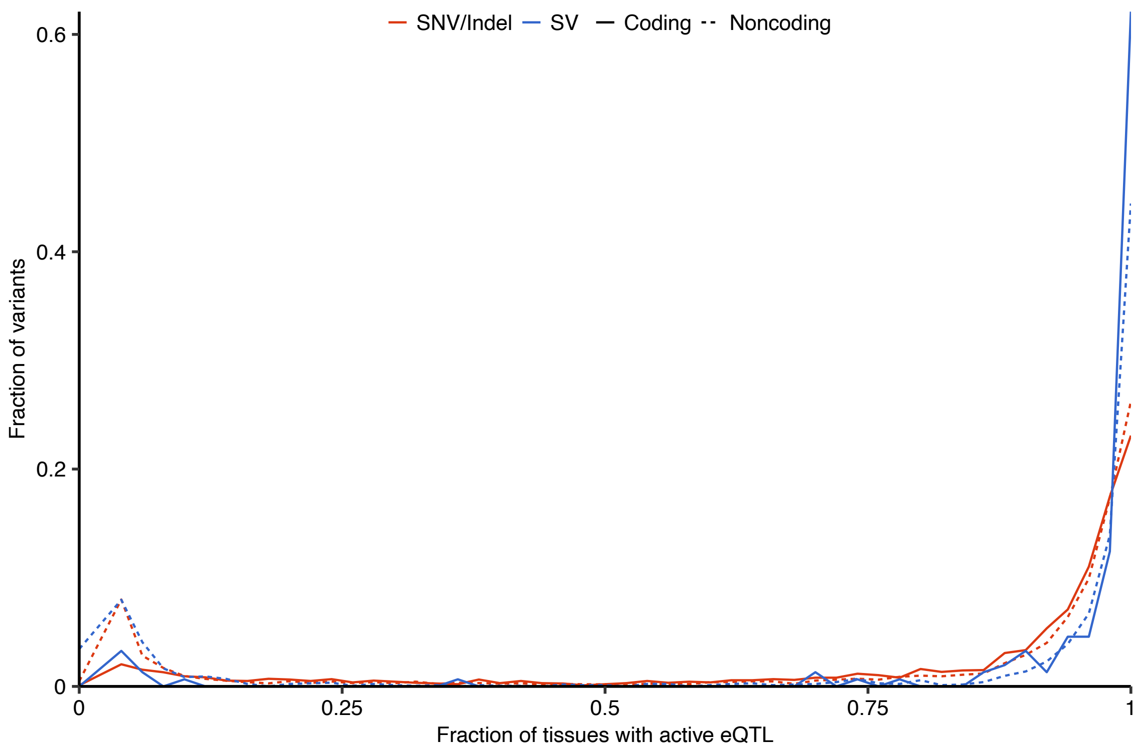

Supplemental Fig. S5. Distribution of the tissue specificity of eQTLs across tissues, as evaluated by METASOFT, for eQTLs in which the activity status is known in at least 43 of 48 evaluated tissues. Red lines indicate the distribution of SV-eQTLs that are active in the fraction of evaluated tissues indicated on the x-axis. Blue lines indicate the same for SNV- and indel-eQTLs. Solid lines denote coding eQTLs where the eVariant intersects the coding region of the associated eGene and dashed lines show the distributions for noncoding eQTLs.

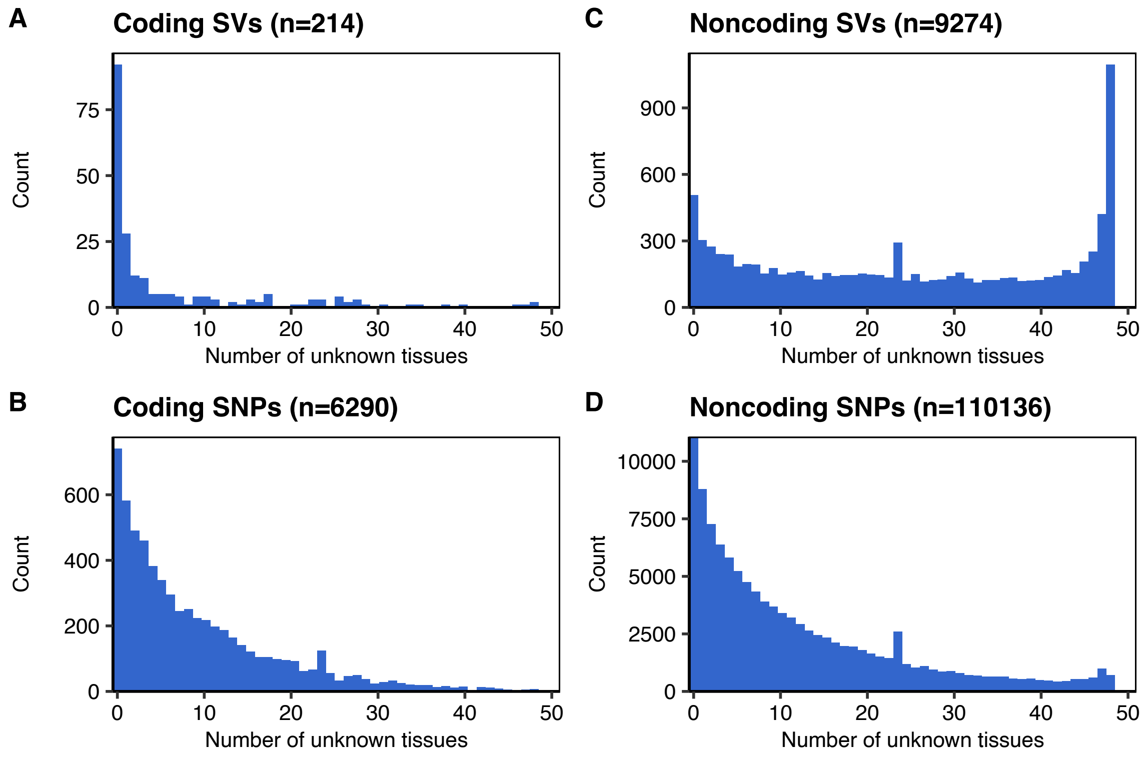

Supplemental Fig. S6. Distribution of eQTLs with unknown status (0.1≤*m*≤0.9) across the indicated number of tissues as evaluated by METASOFT. (**A**) Distribution for coding SV-eQTLs where the eSV intersects with the coding region of the associated eGene. (**B**) Distribution for coding SNV/indel-eQTLs. (**C**) Distribution for noncoding SV-eQTLs where the eSV does not intersect the coding region of the associated eGene. A large number of noncoding SV-eQTLs (1,094/9,274) have unknown status in all 48 tissues evaluated. (**D**) Distribution for noncoding SNV/indel-eQTLs**.**

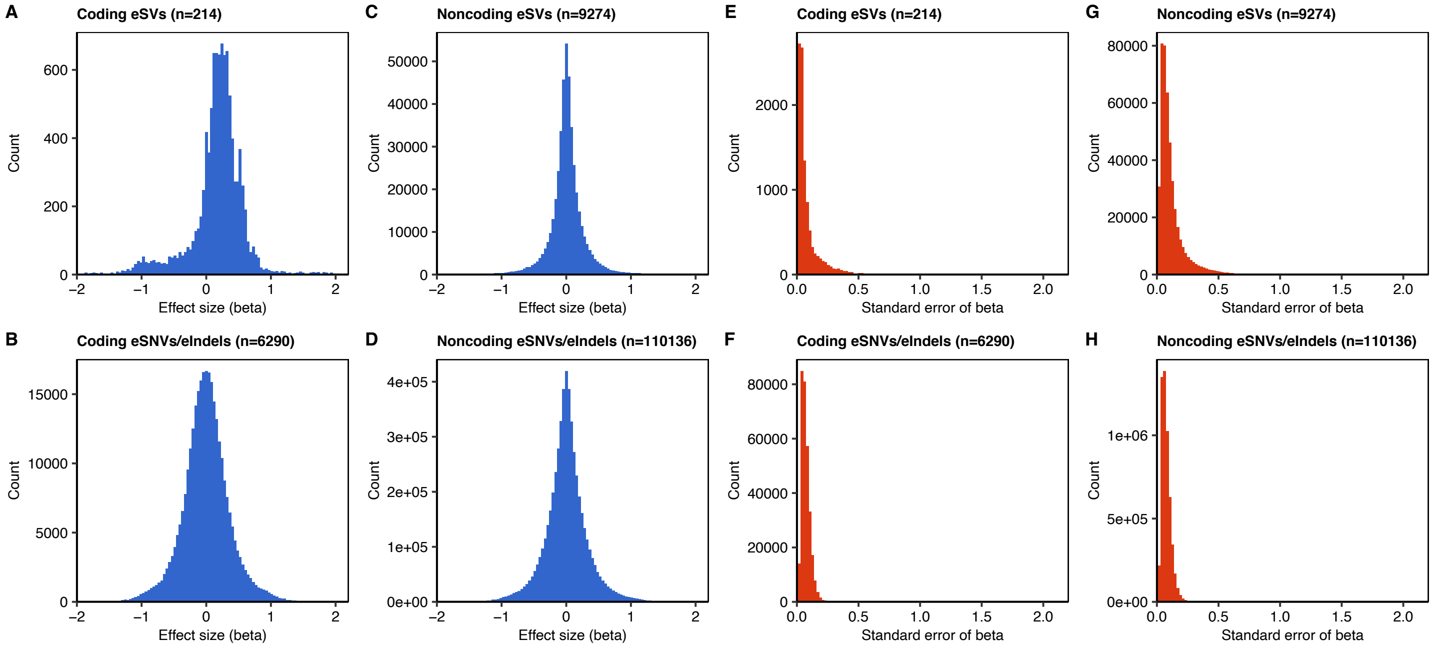

Supplemental Fig. S7. Distribution of eQTL effect sizes and the standard errors of effect sizes for eQTLs evaluated by METASOFT. (**A-D**) Distribution of eQTL effect sizes (beta) for all eQTLs evaluated by METASOFT across all 48 tissues. Coding eSVs (**A**) have much larger effect sizes compared to coding eSNVs and eIndels (**B**), noncoding eSVs (**C**) and noncoding eSNVs/eIndels (**D**). (**E-H**) Distribution of eQTL effect size standard errors for all eQTLs evaluated by METASOFT across all 48 tissues. Both coding eSVs (**E**) and noncoding eSVs (**G**) have larger standard errors compared to coding eSNVs/eIndels (**F**) and noncoding eSNVs/eIndels (**G**).

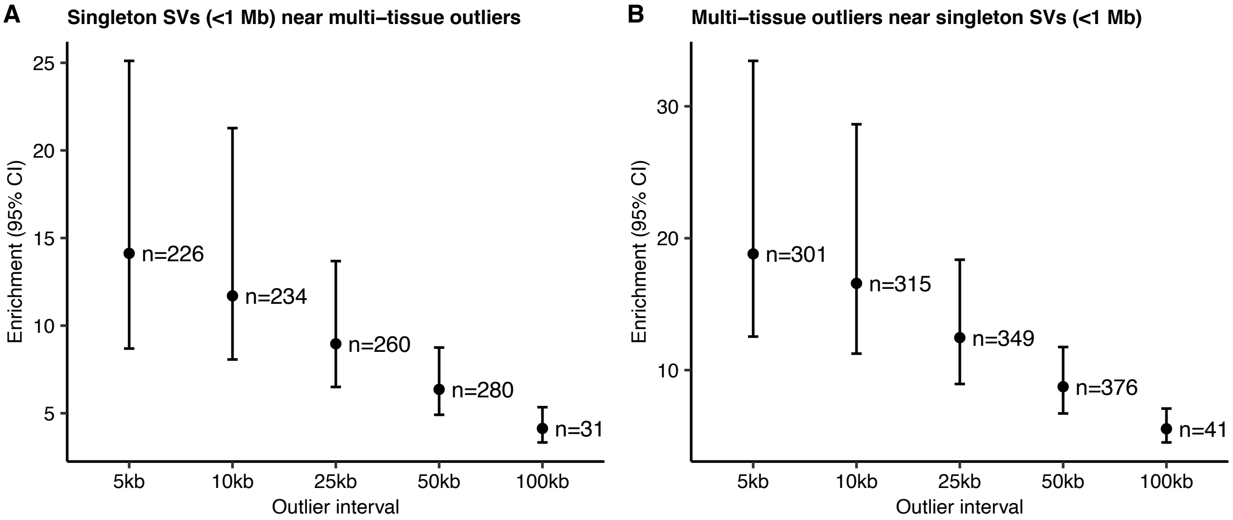

Supplemental Fig. S8. Fold enrichment of European singleton SVs within the indicated distance of multi-tissue expression outliers (**A**) and fold enrichment of multi-tissue outliers within indicated distance of European singleton SVs (**B**). Enrichments calculated between the observed set of 26,289 autosomal multi-tissue outliers and 1,000 random permutations of outlier sample names. All included SVs are smaller than 1 Mb in size. Only European ancestry samples were included in this analysis. 95% confidence intervals are indicated by the error bars and $p<0.001$ for the enrichment at all distances.

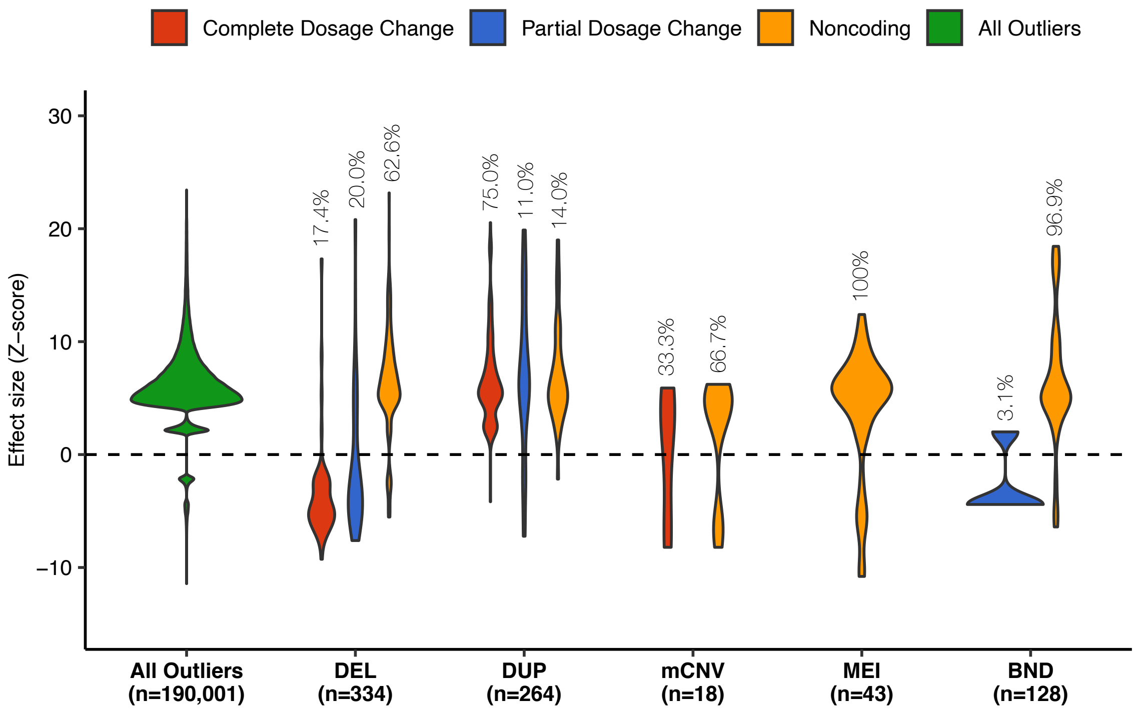

Supplemental Fig. S9. Distribution of gene expression outlier effect sizes. The effect sizes for all outliers are shown in green. Outliers associated with each SV type are also shown, separated by whether the SV causes a complete dosage change (red), partial dosage change (blue) or is noncoding for the associated outlier gene (yellow). Effect sizes shown are the most extreme effect size for each outlier gene across all tissues with available expression data. Counts below the x-axis indicate the number of unique SV/outlier pairs with the indicated SV type. Percentages above distributions indicate the fraction of SV/outlier pairs with the relevant variant type found in each dosage category.

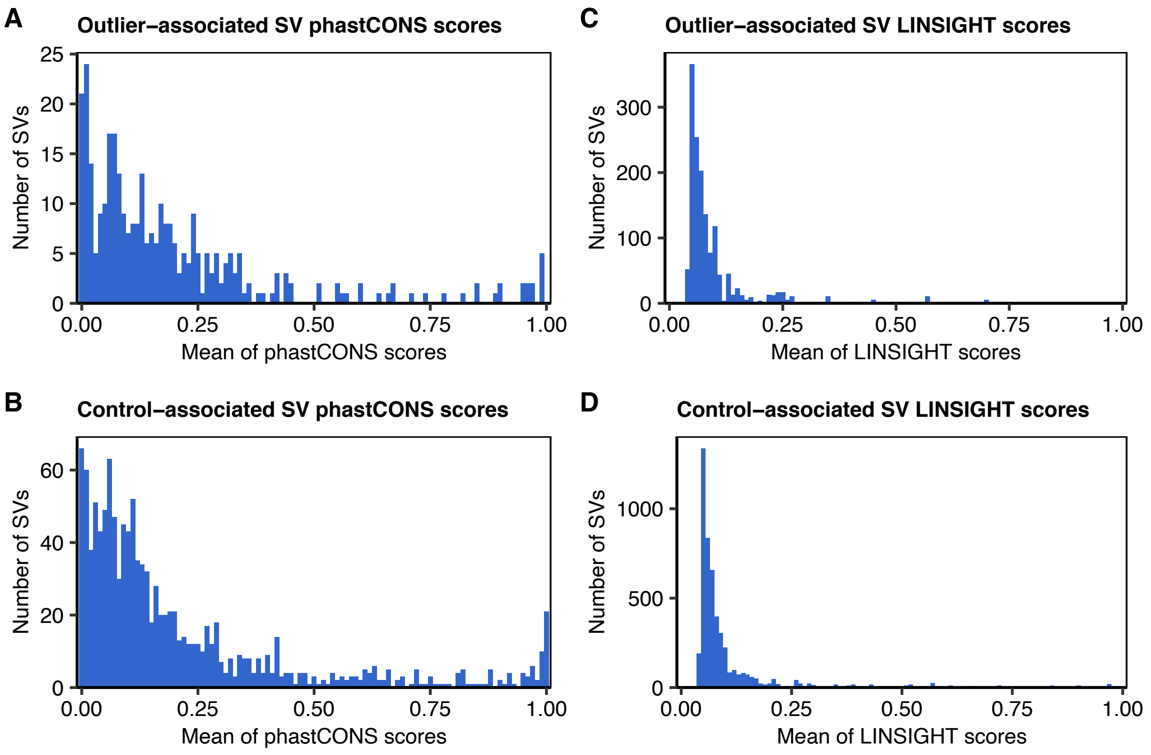

Supplemental Fig. S10. Distribution of SV impact scores calculated with SVScore. (**A-B**) Distribution of mean phastCONS scores for outlier-associated (**A**) and control-associated (**B**) SVs. (**C-D**) Distribution of mean LINSIGHT scores for outlier-associated (**C**) and control-associated (**D**) SVs.

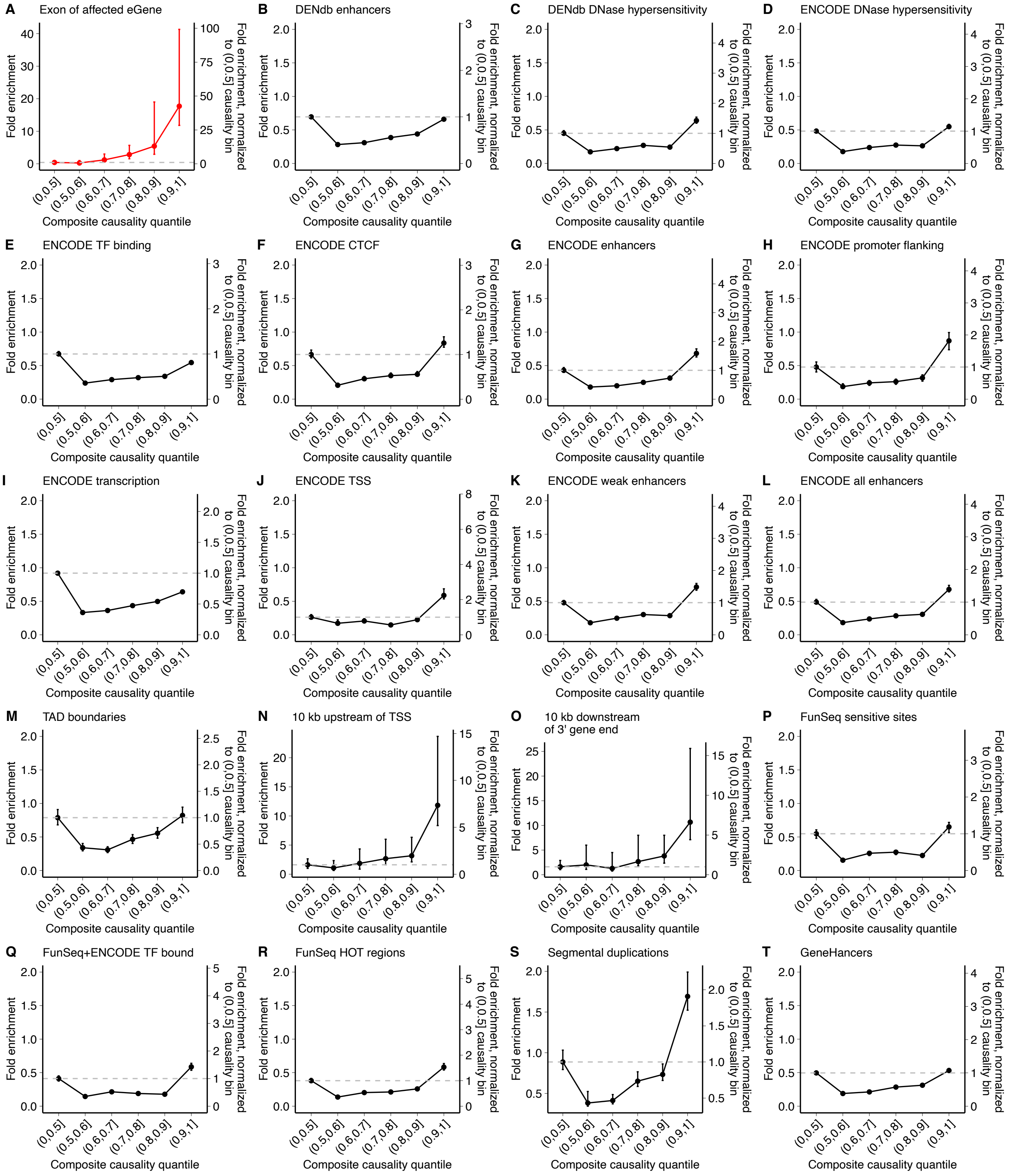

Supplemental Fig. S11. Feature enrichment of SV-eQTLs repeated with no additional padding around any features. Fold enrichment and 95% confidence intervals (based on 100 random shuffled sets of the positions of SVs in each bin) for the overlap between the most significant SV for each eGene and various annotated genomic features. (**A**) Enrichment of SVs in each causality bin for intersections with exons of the affected eGene. (**B-T**) For the remaining plots, SVs that overlapped with an exon of the affected eGenes were excluded. As in **Supplemental Fig. S3**, enrichment was only observed in the 10-kb regions upstream (**N**) and downstream (**O**) of TSSs and in segmental duplications (**S**).
